# Supplementary material for: How Is Spinal Cord Function Measured in Degenerative Cervical Myelopathy? A Systematic Review
Source: J Clin Med. 2022 Mar 5;11(5):1441. doi: 10.3390/jcm11051441 (PMC8910882; doi:10.3390/jcm11051441)
Supplement: Supplementary file 1 [file jcm-11-01441-s001.zip › jcm-1582747 - supplementary material S1.pdf]

Supplementary Materials S1. Electronic database search terms; Supplementary Material

Pubmed (MEDLINE) Search Strategy

(((magnetic resonance imaging) OR (MRI)) AND (((((((SCI) OR (spinal cord injury)) OR (spinal trauma)) OR (spine fracture)) OR (spine trauma)) OR (cervical fracture)) OR (cervical trauma))) AND ((((((((outcome) OR (recovery)) OR (management)) OR (decision-making)) OR (decision)) OR (surgery)) OR (surgical)) OR (treatment))

Embase Ovid Search Strategy

(magnetic AND resonance AND imaging OR mri) AND (((((((sci OR spinal) AND cord AND injury OR spinal) AND trauma OR spine) AND fracture OR spine) AND trauma OR cervical) AND fracture OR cervical) AND trauma) AND ((outcome OR recovery OR management OR decision) AND making OR decision OR surgery OR surgical OR treatment)

CENTRAL Search Strategy

[Magnetic Resonance Imaging] explode all trees OR MRI AND [Spinal Cord Injuries] explode all trees OR spinal cord injury OR SCI OR spinal trauma OR spine trauma OR spine fracture OR cervical fracture OR cervical trauma AND [Outcome Assessment, Health Care] explode all trees OR outcome OR recovery OR management OR MeSH descriptor: [Clinical Decision-Making] explode all trees OR decision-making OR decision OR MeSH descriptor: [General Surgery] explode all trees OR surgery OR surgical OR treatment OR MeSH descriptor: [Therapeutics] explode all trees
