# Supplementary material for: How Is Spinal Cord Function Measured in Degenerative Cervical Myelopathy? A Systematic Review
Source: J Clin Med. 2022 Mar 5;11(5):1441. doi: 10.3390/jcm11051441 (PMC8910882; doi:10.3390/jcm11051441)
Supplement: Supplementary file 1 [file jcm-11-01441-s001.zip › jcm-1582747 - supplementary material S2.pdf]

Supplementary Materials S2. Reference list of studies included in systematic review

Included Studies from Systematic Review (APA 6th formatted):

1. Al-Tamimi, Y. Z., Guilfoyle, M., Seeley, H., & Laing, R. J. (2013). Measurement of long-term outcome in patients with cervical spondylotic myelopathy treated surgically. Eur Spine J, 22(11), 2552-2557. doi:10.1007/s00586-013-2965-4
2. Allam, A. F. A., Abotakia, T. A. A., & Koptan, W. (2018). Role of Cerebrolysin in cervical spondylotic myelopathy patients: a prospective randomized study. Spine J, 18(7), 1136-1142. doi:10.1016/j.spinee.2017.11.002
3. Arnold, Paul M., MD, Branko Kopjar, MD, Lindsay Tetreault, BSc, Hiroaki Nakashima, MD, Michael G. Fehlings, MD, PhD, FRCS(C), FACS, 162 Tobacco Smoking and Outcomes of Surgical Decompression in Patients With Symptomatic Degenerative Cervical Spondylotic Myelopathy, Neurosurgery, Volume 63, Issue CN_suppl_1, August 2016, Page 165, https://doi.org/10.1227/01.neu.0000489731.76982.a4
4. Arnold, P. M., Fehlings, M. G., Kopjar, B., Yoon, S. T., Massicotte, E. M., Vaccaro, A. R., Gokaslan, Z. L. (2014). Mild diabetes is not a contraindication for surgical decompression in cervical spondylotic myelopathy: results of the AOSpine North America multicenter prospective study (CSM). Spine J, 14(1), 65-72. doi:10.1016/j.spinee.2013.06.016
5. Asher, A. L., Devin, C. J., Kerezoudis, P., Chotai, S., Nian, H., Harrell, F. E., Bydon, M. (2019). Comparison of Outcomes Following Anterior vs Posterior Fusion Surgery for Patients With Degenerative Cervical Myelopathy: An Analysis From Quality Outcomes Database. Neurosurgery, 84(4), 919-926. doi:10.1093/neuros/nyy144
6. Badhiwala, J. H., Hachem, L. D., Merali, Z., Witiw, C. D., Nassiri, F., Akbar, M. A., Fehlings, M. G. (2020). Predicting Outcomes After Surgical Decompression for Mild Degenerative Cervical Myelopathy: Moving Beyond the mJOA to Identify Surgical Candidates. Neurosurgery, 86(4), 565-573. doi:10.1093/neuros/nyz160
7. Badhiwala, J. H., Khan, O., Wegner, A., Jiang, F., Wilson, J. R. F., Morgan, B. R., Fehlings, M. G. (2020). A partial least squares analysis of functional status, disability, and quality of life after surgical decompression for degenerative cervical myelopathy. Sci Rep, 10(1), 16132. doi:10.1038/s41598-020-72595-2
8. Badhiwala, J. H., Witiw, C. D., Nassiri, F., Akbar, M. A., Jaja, B., Wilson, J. R., & Fehlings, M. G. (2018). Minimum Clinically Important Difference in SF-36 Scores for Use in Degenerative Cervical Myelopathy. Spine (Phila Pa 1976), 43(21), E1260-E1266. doi:10.1097/BRS.0000000000002684
9. Badhiwala, J. H., Witiw, C. D., Nassiri, F., Akbar, M. A., Mansouri, A., Wilson, J. R., & Fehlings, M. G. (2019). Efficacy and Safety of Surgery for Mild Degenerative Cervical Myelopathy: Results of the AOSpine North America and International Prospective Multicenter Studies. Neurosurgery, 84(4), 890-897. doi:10.1093/neuros/nyy133
10. Badhiwala, J. H., Witiw, C. D., Nassiri, F., Jaja, B. N. R., Akbar, M. A., Mansouri, A., Fehlings, M. G. (2018). Patient phenotypes associated with outcome following surgery for mild degenerative cervical myelopathy: a principal component regression analysis. Spine J, 18(12), 2220-2231. doi:10.1016/j.spinee.2018.05.009
11. Bapat, M. R., Chaudhary, K., Sharma, A., & Laheri, V. (2008). Surgical approach to cervical spondylotic myelopathy on the basis of radiological patterns of compression: prospective analysis of 129 cases. Eur Spine J, 17(12), 1651-1663. doi:10.1007/s00586-008-0792-9
12. Buchowski, J. M., Anderson, P. A., Sekhon, L., & Riew, K. D. (2009). Cervical disc arthroplasty compared with arthrodesis for the treatment of myelopathy. Surgical technique. J Bone Joint Surg Am, 91 Suppl 2, 223-232. doi:10.2106/JBJS.I.00564
13. Butler, M. B., Mowforth, O. D., Badran, A., Starkey, M., Boerger, T., Sadler, I., Kotter, M. R. N. (2020). Provision and Perception of Physiotherapy in the Nonoperative Management of Degenerative Cervical Myelopathy (DCM): A Cross-Sectional Questionnaire of People Living With DCM. Global Spine J, 2192568220961357. doi:10.1177/2192568220961357
14. Chen, H., Liu, H., Meng, Y., Wang, B., Gong, Q., & Song, Y. (2018). Short-Term Outcomes of Anterior Fusion-Nonfusion Hybrid Surgery versus Posterior Cervical Laminoplasty in the Treatment of Multilevel Cervical Spondylotic Myelopathy. World neurosurgery, 116, e1007–e1014. https://doi.org/10.1016/j.wneu.2018.05.150
15. Chen, E. L., Wang, N., & Quan, R. F. (2020). Zhongguo gu shang = China journal of orthopaedics and traumatology, 33(9), 841–847. https://doi.org/10.12200/j.issn.1003-0034.2020.09.010
16. Davies, B. M., Munro, C., Khan, D. Z., Fitzpatrick, S. M., Hilton, B., Mowforth, O. D., Kotter, M. R. N. (2020). Outcomes of Degenerative Cervical Myelopathy From The Perspective of Persons Living With the Condition: Findings of a Semistructured Interview Process With Partnered Internet Survey. Global Spine J, 2192568220953811. doi:10.1177/2192568220953811

17. Davies, B. M., Nourallah, B., Venkatesh, A., Ali, A. M. S., Guilfoyle, M., Francis, J., Laing, R. J. C. (2020). Establishing mild, moderate and severe criteria for the myelopathy disability index in cervical spondylotic myelopathy. Br J Neurosurg, 1-5. doi:10.1080/02688697.2020.1839741
18. Deng, B. & LI, J. & Zhang, YI & Teng, Y.. (2021). Comparison of Three Different Surgical Methods in the Treatment of Cervical Spondylotic Myelopathy. Indian Journal of Pharmaceutical Sciences. 83. 10.36468/pharmaceutical-sciences.spl.169.
19. Eryilmaz, F., & Farooque, U. (2021). The Efficacy of Combined Medication With Methylprednisolone and Erythropoietin in the Treatment of Ischemia-Reperfusion Injury to the Spinal Cord in Patients With Cervical Spondylotic Myelopathy. Cureus, 13(3), e14018. doi:10.7759/cureus.14018
20. Evaniew, N., Cadotte, D. W., Dea, N., Bailey, C. S., Christie, S. D., Fisher, C. G., Jacobs, W. B. (2020). Clinical predictors of achieving the minimal clinically important difference after surgery for cervical spondylotic myelopathy: an external validation study from the Canadian Spine Outcomes and Research Network. J Neurosurg Spine, 1-9. doi:10.3171/2020.2.SPINE191495
21. Fehlings, M. G., Badhiwala, J. H., Ahn, H., Farhadi, H. F., Shaffrey, C. I., Nassr, A., Kopjar, B. (2021). Safety and efficacy of riluzole in patients undergoing decompressive surgery for degenerative cervical myelopathy (CSM-Protect): a multicentre, double-blind, placebo-controlled, randomised, phase 3 trial. Lancet Neurol, 20(2), 98-106. doi:10.1016/S1474-4422(20)30407-5
22. Fehlings, M. G., Barry, S., Kopjar, B., Yoon, S. T., Arnold, P., Massicotte, E. M., Gokaslan, Z. L. (2013a). Anterior versus posterior surgical approaches to treat cervical spondylotic myelopathy: outcomes of the prospective multicenter AOSpine North America CSM study in 264 patients. Spine (Phila Pa 1976), 38(26), 2247-2252. doi:10.1097/BRS.0000000000000047
23. Fehlings, M. G., Ibrahim, A., Tetreault, L., Albanese, V., Alvarado, M., Arnold, P., Kopjar, B. (2015). A global perspective on the outcomes of surgical decompression in patients with cervical spondylotic myelopathy: results from the prospective multicenter AOSpine international study on 479 patients. Spine (Phila Pa 1976), 40(17), 1322-1328. doi:10.1097/BRS.0000000000000988
24. Fehlings, M. G., Kopjar, B., Ibrahim, A., Tetreault, L. A., Arnold, P. M., Defino, H., Dekutoski, M. B. (2018). Geographic variations in clinical presentation and outcomes of decompressive surgery in patients with symptomatic degenerative cervical myelopathy: analysis of a prospective, international multicenter cohort study of 757 patients. Spine J, 18(4), 593-605. doi:10.1016/j.spinee.2017.08.265
25. Fehlings, M. G., Santaguida, C., Tetreault, L., Arnold, P., Barbagallo, G., Defino, H., Kopjar, B. (2017). Laminectomy and fusion versus laminoplasty for the treatment of degenerative cervical myelopathy: results from the AOSpine North America and International prospective multicenter studies. Spine J, 17(1), 102-108. doi:10.1016/j.spinee.2016.08.019
26. Fehlings, M. G., Smith, J. S., Kopjar, B., Arnold, P. M., Yoon, S. T., Vaccaro, A. R., Shaffrey, C. I. (2012a). Perioperative and delayed complications associated with the surgical treatment of cervical spondylotic myelopathy based on 302 patients from the AOSpine North America Cervical Spondylotic Myelopathy Study. J Neurosurg Spine, 16(5), 425-432. doi:10.3171/2012.1.SPINE11467
27. Fehlings, M. G., Wilson, J. R., Kopjar, B., Yoon, S. T., Arnold, P. M., Massicotte, E. M., Gokaslan, Z. L. (2013a). Efficacy and safety of surgical decompression in patients with cervical spondylotic myelopathy: results of the AOSpine North America prospective multi-center study. J Bone Joint Surg Am, 95(18), 1651-1658. doi:10.2106/JBJS.L.00589
28. Funaba, M., Kanchiku, T., Yoshida, G., Imagama, S., Kawabata, S., Fujiwara, Y., & Matsuyama, Y. (2022). Efficacy of Intraoperative Neuromonitoring Using Transcranial Motor-Evoked Potentials for Degenerative Cervical Myelopathy: A Prospective Multicenter Study by the Monitoring Committee of the Japanese Society for Spine Surgery and Related Research. Spine, 47(1), E27-E37.
29. Gao, S. J., Yuan, X., Jiang, X. Y., Liu, X. X., Liu, X. P., Wang, Y. F., Xu, K. (2013). Correlation study of 3T-MR-DTI measurements and clinical symptoms of cervical spondylotic myelopathy. Eur J Radiol, 82(11), 1940-1945. doi:10.1016/j.ejrad.2013.06.011
30. Ghogawala, Z., Benzel, E. C., Heary, R. F., Riew, K. D., Albert, T. J., Butler, W. E., Schwartz, J. S. (2014). Cervical spondylotic myelopathy surgical trial: randomized, controlled trial design and rationale. Neurosurgery, 75(4), 334-346. doi:10.1227/NEU.0000000000000479
31. Ghogawala, Z., Terrin, N., Dunbar, M. R., Breeze, J. L., Freund, K. M., Kanter, A. S., Benzel, E. C. (2021). Effect of Ventral vs Dorsal Spinal Surgery on Patient-Reported Physical Functioning in Patients With Cervical Spondylotic Myelopathy: A Randomized Clinical Trial. JAMA, 325(10), 942-951. doi:10.1001/jama.2021.1233

32. Hartig, D., Batke, J., Dea, N., Kelly, A., Fisher, C., & Street, J. (2015). Adverse events in surgically treated cervical spondylopathic myelopathy: a prospective validated observational study. Spine (Phila Pa 1976), 40(5), 292-298. doi:10.1097/BRS.0000000000000755
33. Hirai, T., Nishimura, S., Yoshii, T., Nagoshi, N., Hashimoto, J., Mori, K., Kawaguchi, Y. (2021). Associations between Clinical Findings and Severity of Diffuse Idiopathic Skeletal Hyperostosis in Patients with Ossification of the Posterior Longitudinal Ligament. J Clin Med, 10(18). doi:10.3390/jcm10184137
34. Hirai, T., Yoshii, T., Egawa, S., Sakai, K., Kusano, K., Nakagawa, Y., Okawa, A. (2021). Severity of Myelopathy is Closely Associated With Advanced Age and Signal Intensity Change in Cervical Ossification of the Posterior Longitudinal Ligament: A Prospective Nationwide Investigation. Clin Spine Surg. doi:10.1097/BSD.0000000000001164
35. Hirai, T., Yoshii, T., Ushio, S., Hashimoto, J., Mori, K., Maki, S., Kawaguchi, Y. (2020). Associations between Clinical Symptoms and Degree of Ossification in Patients with Cervical Ossification of the Posterior Longitudinal Ligament: A Prospective Multi-Institutional Cross-Sectional Study. J Clin Med, 9(12). doi:10.3390/jcm9124055
36. Hirai, T., Yoshii, T., Ushio, S., Mori, K., Maki, S., Katsumi, K., Kawaguchi, Y. (2020). Clinical characteristics in patients with ossification of the posterior longitudinal ligament: A prospective multi-institutional cross-sectional study. Sci Rep, 10(1), 5532. doi:10.1038/s41598-020-62278-3
37. Hou, Y., Liang, L., Shi, G. D., Xu, P., Xu, G. H., Shi, J. G., & Yuan, W. (2017). Comparing effects of cervical anterior approach and laminoplasty in surgical management of cervical ossification of posterior longitudinal ligament by a prospective nonrandomized controlled study. Orthop Traumatol Surg Res, 103(5), 733-740. doi:10.1016/j.otsr.2017.05.011
38. Imagama, S., Ando, K., Takeuchi, K., Kato, S., Murakami, H., Aizawa, T., Okawa, A. (2018). Perioperative Complications After Surgery for Thoracic Ossification of Posterior Longitudinal Ligament: A Nationwide Multicenter Prospective Study. Spine (Phila Pa 1976), 43(23), E1389-E1397. doi:10.1097/BRS.0000000000002703
39. Inose, H., Yoshii, T., Kimura, A., Takeshita, K., Inoue, H., Maekawa, A., Okawa, A. (2020). Comparison of Clinical and Radiographic Outcomes of Laminoplasty, Anterior Decompression With Fusion, and Posterior Decompression With Fusion for Degenerative Cervical Myelopathy: A Prospective Multicenter Study. Spine (Phila Pa 1976), 45(20), E1342-E1348. doi:10.1097/BRS.0000000000003592
40. Inose, H., Yoshii, T., Kimura, A., Takeshita, K., Inoue, H., Maekawa, A., Okawa, A. (2021). Predictors of Falls in Patients with Degenerative Cervical Myelopathy: A Prospective Multi-institutional Study. Spine (Phila Pa 1976), 46(15), 1007-1013. doi:10.1097/BRS.0000000000003958
41. Inoue, T., Soshi, S., Kubota, M., & Marumo, K. (2020). Efficacy of Laminoplasty in Improving Sensory Disturbances in Patients with Cervical Spondylotic Myelopathy: A Prospective Study. World Neurosurg, 134, e581-e588. doi:10.1016/j.wneu.2019.10.141
42. Ito, K., Imagama, S., Ito, Z., Ando, K., Kobayashi, K., Hida, T., Kato, F. (2017). MRI Signal Intensity Classification in Cervical Ossification of the Posterior Longitudinal Ligament: Predictor of Surgical Outcomes. Spine (Phila Pa 1976), 42(2), E98-E103. doi:10.1097/BRS.0000000000001717
43. Ito, K., Yukawa, Y., Machino, M., Kanbara, S., Nakashima, H., Hida, T., Kato, F. (2015). Dynamic changes in the spinal cord cross-sectional area in patients with myelopathy due to cervical ossification of posterior longitudinal ligament. Spine J, 15(3), 461-466. doi:10.1016/j.spinee.2014.10.001
44. Jeffreys, R. V. (1986). The surgical treatment of cervical myelopathy due to spondylosis and disc degeneration. J Neurol Neurosurg Psychiatry, 49(4), 353-361. doi:10.1136/jnnp.49.4.353
45. Jeyamohan, S. B., Kenning, T. J., Petronis, K. A., Feustel, P. J., Drazin, D., & DiRisio, D. J. (2015). Effect of steroid use in anterior cervical discectomy and fusion: a randomized controlled trial. J Neurosurg Spine, 23(2), 137-143. doi:10.3171/2014.12.SPINE14477
46. Kadanka, Z., Adamova, B., Kerkovsky, M., Dusek, L., Jurova, B., Vlckova, E., & Bednarik, J. (2017). Predictors of symptomatic myelopathy in degenerative cervical spinal cord compression. Brain Behav, 7(9), e00797. doi:10.1002/brb3.797
47. Kadanka, Z., Kerkovsky, M., Bednarik, J., & Jarkovsky, J. (2007). Cross-sectional transverse area and hyperintensities on magnetic resonance imaging in relation to the clinical picture in cervical spondylotic myelopathy. Spine (Phila Pa 1976), 32(23), 2573-2577. doi:10.1097/BRS.0b013e318158cda0
48. Kalsi-Ryan, S., Clout, J., Rostami, P., Massicotte, E. M., & Fehlings, M. G. (2019). Duration of symptoms in the quantification of upper limb disability and impairment for individuals with mild degenerative cervical myelopathy (DCM). PLoS One, 14(9), e0222134. doi:10.1371/journal.pone.0222134
49. Kalsi-Ryan, S., Riehm, L. E., Tetreault, L., Martin, A. R., Teoderascu, F., Massicotte, E., Fehlings, M. G. (2020). Characteristics of Upper Limb Impairment Related to Degenerative Cervical Myelopathy: Development of a Sensitive

Hand Assessment (Graded Redefined Assessment of Strength, Sensibility, and Prehension Version Myelopathy). Neurosurgery, 86(3), E292-E299. doi:10.1093/neuros/nyz499
50. Kalsi-Ryan, S., Rienmueller, A. C., Riehm, L., Chan, C., Jin, D., Martin, A. R., Fehlings, M. G. (2020). Quantitative Assessment of Gait Characteristics in Degenerative Cervical Myelopathy: A Prospective Clinical Study. J Clin Med, 9(3). doi:10.3390/jcm9030752
51. Karpova, A., Arun, R., Kalsi-Ryan, S., Massicotte, E. M., Kopjar, B., & Fehlings, M. G. (2014). Do quantitative magnetic resonance imaging parameters correlate with the clinical presentation and functional outcomes after surgery in cervical spondylotic myelopathy? A prospective multicenter study. Spine (Phila Pa 1976), 39(18), 1488-1497. doi:10.1097/BRS.0000000000000436
52. Kato, S., Nouri, A., Reihani-Kermani, H., Oshima, Y., Cheng, J., & Fehlings, M. G. (2018). Postoperative Resolution of Magnetic Resonance Imaging Signal Intensity Changes and the Associated Impact on Outcomes in Degenerative Cervical Myelopathy: Analysis of a Global Cohort of Patients. Spine (Phila Pa 1976), 43(12), 824-831. doi:10.1097/BRS.0000000000002426
53. Kato, S., Nouri, A., Wu, D., Nori, S., Tetreault, L., & Fehlings, M. G. (2017). Comparison of Anterior and Posterior Surgery for Degenerative Cervical Myelopathy: An MRI-Based Propensity-Score-Matched Analysis Using Data from the Prospective Multicenter AOSpine CSM North America and International Studies. J Bone Joint Surg Am, 99(12), 1013-1021. doi:10.2106/JBJS.16.00882
54. Kato, S., Nouri, A., Wu, D., Nori, S., Tetreault, L., & Fehlings, M. G. (2018). Impact of Cervical Spine Deformity on Preoperative Disease Severity and Postoperative Outcomes Following Fusion Surgery for Degenerative Cervical Myelopathy: Sub-analysis of AOSpine North America and International Studies. Spine (Phila Pa 1976), 43(4), 248-254. doi:10.1097/BRS.0000000000002307
55. Katsumi, K., Hirai, T., Yoshii, T., Maki, S., Mori, K., Nagoshi, N., Kawaguchi, Y. (2021). The impact of ossification spread on cervical spine function in patients with ossification of the posterior longitudinal ligament. Sci Rep, 11(1), 14337. doi:10.1038/s41598-021-93602-0
56. Kawakami, M., Tamaki, T., Iwasaki, H., Yoshida, M., Ando, M., & Yamada, H. (2000). A comparative study of surgical approaches for cervical compressive myelopathy. Clin Orthop Relat Res(381), 129-136. doi:10.1097/00003086-200012000-00016
57. Kim, T. H., Ha, Y., Shin, J. J., Cho, Y. E., Lee, J. H., & Cho, W. H. (2016). Signal intensity ratio on magnetic resonance imaging as a prognostic factor in patients with cervical compressive myelopathy. Medicine (Baltimore), 95(39), e4649. doi:10.1097/MD.0000000000004649
58. Kimura, A., Endo, T., Inoue, H., & Seichi, A. (2014). Preoperative predictors of patient satisfaction with outcome after cervical laminoplasty. Global Spine J, 4(2), 77-82. doi:10.1055/s-0034-1366973
59. Kimura, A., Takeshita, K., Inoue, H., Inose, H., Yoshii, T., Maekawa, A., Okawa, A. (2021). The 5-question Geriatric Locomotive Function Scale predicts postoperative fall risk in patients undergoing surgery for degenerative cervical myelopathy. J Orthop Sci, 26(5), 779-785. doi:10.1016/j.jos.2020.08.017
60. Kimura, A., Takeshita, K., Shiraishi, Y., Inose, H., Yoshii, T., Maekawa, A., Okawa, A. (2020). Effectiveness of Surgical Treatment for Degenerative Cervical Myelopathy in Preventing Falls and Fall-related Neurological Deterioration: A Prospective Multi-institutional Study. Spine (Phila Pa 1976), 45(11), E631-E638. doi:10.1097/BRS.0000000000003355
61. Kimura, A., Takeshita, K., Yoshii, T., Egawa, S., Hirai, T., Sakai, K., Okawa, A. (2021). Impact of Diabetes Mellitus on Cervical Spine Surgery for Ossification of the Posterior Longitudinal Ligament. J Clin Med, 10(15). doi:10.3390/jcm10153375
62. Koda, M., Yoshii, T., Egawa, S., Sakai, K., Kusano, K., Nakagawa, Y., Yamazaki, M. (2021a). Factors Significantly Associated with Postoperative Neck Pain Deterioration after Surgery for Cervical Ossification of the Posterior Longitudinal Ligament: Study of a Cohort Using a Prospective Registry. J Clin Med, 10(21). doi:10.3390/jcm10215026
63. Koda, M., Yoshii, T., Egawa, S., Sakai, K., Kusano, K., Nakagawa, Y., Yamazaki, M. (2021b). Neurological improvement is associated with neck pain attenuation after surgery for cervical ossification of the posterior longitudinal ligament. Sci Rep, 11(1), 11910. doi:10.1038/s41598-021-91268-2
64. Kopjar, B., Bohm, P. E., Arnold, J. H., Fehlings, M. G., Tetreault, L. A., & Arnold, P. M. (2018). Outcomes of Surgical Decompression in Patients With Very Severe Degenerative Cervical Myelopathy. Spine (Phila Pa 1976), 43(16), 1102-1109. doi:10.1097/BRS.0000000000002602
65. Kopjar, B., Tetreault, L., Kalsi-Ryan, S., & Fehlings, M. (2015). Psychometric properties of the modified Japanese Orthopaedic Association scale in patients with cervical spondylotic myelopathy. Spine (Phila Pa 1976), 40(1), E23-28. doi:10.1097/BRS.0000000000000648

66. Kotter, M. R. N., Tetreault, L., Badhiwala, J. H., Wilson, J. R., Arnold, P. M., Bartels, R., Fehlings, M. G. (2020). Surgical Outcomes Following Laminectomy With Fusion Versus Laminectomy Alone in Patients With Degenerative Cervical Myelopathy. Spine (Phila Pa 1976), 45(24), 1696-1703. doi:10.1097/BRS.0000000000003677
67. Lee, Y. S., Cho, D. C., Sung, J. K., Han, I., Kim, C. H., Kim, J. Y., & Kim, K. T. (2020). The Effect of an Educational and Interactive Informed Consent Process on Patients With Cervical Spondylotic Myelopathy Caused by Ossification of the Posterior Longitudinal Ligament. Spine (Phila Pa 1976), 45(3), 193-200. doi:10.1097/BRS.0000000000003223
68. Li, P., Wei, Z., Zhang, H., Zhang, K., & Li, J. (2019). Effects of decompressive operation on cardiac autonomic regulation in patients with cervical spondylotic myelopathy: analysis of blood pressure, heart rate, and heart rate variability. Eur Spine J, 28(8), 1864-1871. doi:10.1007/s00586-019-05972-9
69. Li, P., Zhang, H., Cheng, H., Meng, F., & Li, J. (2019). Recovery Process After Anterior Cervical Decompression in Patients With Cervical Spondylotic Myelopathy With Different Natural History. Clin Spine Surg, 32(8), 337-344. doi:10.1097/BSD.0000000000000873
70. Li, S., Kodama, J., Wei, L., Wu, T., Fujiwara, H., Nagamoto, Y., Liu, X. (2021). Japanese Orthopaedic Association Cervical Myelopathy Evaluation Questionnaire as an outcome measure for ossification of posterior longitudinal ligament patients in East Asia: an investigation of reliability, validity, and responsiveness. Ann Transl Med, 9(13), 1060. doi:10.21037/atm-20-8064
71. Li, Y., Gou, L., Bai, Y., Luo, X., Li, W., & Li, L. (2018). Clinical effect of two anterior approaches to adjacent two-segment cervical spondylotic myelopathy. Biomedical Research-tokyo, 29, 1089-1092.
72. Lian, X. F., Xu, J. G., Zeng, B. F., Zhou, W., Kong, W. Q., & Hou, T. S. (2010a). Noncontiguous anterior decompression and fusion for multilevel cervical spondylotic myelopathy: a prospective randomized control clinical study. Eur Spine J, 19(5), 713-719. doi:10.1007/s00586-010-1319-8
73. Lian, X. F., Xu, J. G., Zeng, B. F., Zhou, W., Kong, W. Q., & Hou, T. S. (2010b). Noncontiguous anterior decompression and fusion for multilevel cervical spondylotic myelopathy: a prospective randomized control clinical study. Eur Spine J, 19(5), 713-719. doi:10.1007/s00586-010-1319-8
74. Liu, S., Lafage, R., Smith, J. S., Protopsaltis, T. S., Lafage, V. C., Challier, V., Ames, C. P. (2015). Impact of dynamic alignment, motion, and center of rotation on myelopathy grade and regional disability in cervical spondylotic myelopathy. J Neurosurg Spine, 23(6), 690-700. doi:10.3171/2015.2.SPINE14414
75. Lu, C., Wang, C., Ding, M., Wu, Y., Lv, R., Yuan, J., Shi, Z., & Mao, N. (2018). Posterior surgery may be the best option for the treatment of cervical spondylotic myelopathy: a comparative study in China.
76. Lu, K., Gao, X., Tong, T., Miao, D., Ding, W., & Shen, Y. (2017). Clinical Predictors of Surgical Outcomes and Imaging Features in Single Segmental Cervical Spondylotic Myelopathy with Lower Cervical Instability. Med Sci Monit, 23, 3697-3705. doi:10.12659/msm.906046
77. Machino, M., Ando, K., Kobayashi, K., Ito, K., Tsushima, M., Matsumoto, A., Imagama, S. (2018). The Feature of Clinical and Radiographic Outcomes in Elderly Patients With Cervical Spondylotic Myelopathy: A Prospective Cohort Study on 1025 Patients. Spine (Phila Pa 1976), 43(12), 817-823. doi:10.1097/BRS.0000000000002446
78. Machino, M., Ando, K., Kobayashi, K., Ito, K., Tsushima, M., Morozumi, M., Imagama, S. (2018). Alterations in Intramedullary T2-weighted Increased Signal Intensity following Laminoplasty in Cervical Spondylotic Myelopathy Patients: Comparison Between Pre- and Postoperative Magnetic Resonance Images. Spine (Phila Pa 1976), 43(22), 1595-1601. doi:10.1097/BRS.0000000000002674
79. Machino, M., Ando, K., Kobayashi, K., Nakashima, H., Kanbara, S., Ito, S., Imagama, S. (2020). Prediction of outcome following laminoplasty of cervical spondylotic myelopathy: Focus on the minimum clinically important difference. J Clin Neurosci, 81, 321-327. doi:10.1016/j.jocn.2020.09.065
80. Machino, M., Ando, K., Kobayashi, K., Ota, K., Morozumi, M., Tanaka, S., Imagama, S. (2019). Postoperative Resolution of MR T2 Increased Signal Intensity in Cervical Spondylotic Myelopathy: The Impact of Signal Change Resolution on the Outcomes. Spine (Phila Pa 1976), 44(21), E1241-E1247. doi:10.1097/BRS.0000000000003128
81. Machino, M., Imagama, S., Ando, K., Kobayashi, K., Hida, T., Ito, K., Ishiguro, N. (2017a). Characteristics of Residual Symptoms After Laminoplasty in Diabetic Patients With Cervical Spondylotic Myelopathy: A Prospective Cohort Study. Spine (Phila Pa 1976), 42(12), E708-E715. doi:10.1097/BRS.0000000000001947
82. Machino, M., Imagama, S., Ando, K., Kobayashi, K., Hida, T., Ito, K., Ishiguro, N. (2017b). Prospective Comparison of Age- and Sex-related Differences in Quantifiable 10-S Grip and Release and 10-S Step Test Results for Diagnosis of Cervical Spondylotic Myelopathy in 454 Patients With Cervical Spondylotic Myelopathy and 818 Asymptomatic Subjects. Spine (Phila Pa 1976), 42(8), 578-585. doi:10.1097/BRS.0000000000001849
83. Machino, M., Imagama, S., Ando, K., Kobayashi, K., Ito, K., Tsushima, M., Ishiguro, N. (2018). Image Diagnostic Classification of Magnetic Resonance T2 Increased Signal Intensity in Cervical Spondylotic Myelopathy: Clinical

Evaluation Using Quantitative and Objective Assessment. Spine (Phila Pa 1976), 43(6), 420-426. doi:10.1097/BRS.0000000000002328
84. Machino, M., Yukawa, Y., Hida, T., Ito, K., Nakashima, H., Kanbara, S., Kato, F. (2012). Can elderly patients recover adequately after laminoplasty?: a comparative study of 520 patients with cervical spondylotic myelopathy. Spine (Phila Pa 1976), 37(8), 667-671. doi:10.1097/BRS.0b013e31823147c9
85. Machino, M., Yukawa, Y., Hida, T., Ito, K., Nakashima, H., Kanbara, S., Kato, F. (2013). Modified double-door laminoplasty in managing multilevel cervical spondylotic myelopathy: surgical outcome in 520 patients and technique description. J Spinal Disord Tech, 26(3), 135-140. doi:10.1097/BSD.0b013e31823d848b
86. Machino, M., Yukawa, Y., Imagama, S., Ito, K., Katayama, Y., Matsumoto, T., Kato, F. (2016). Surgical Treatment Assessment of Cervical Laminoplasty Using Quantitative Performance Evaluation in Elderly Patients: A Prospective Comparative Study in 505 Patients With Cervical Spondylotic Myelopathy. Spine (Phila Pa 1976), 41(9), 757-763. doi:10.1097/BRS.0000000000001313
87. Martin, A. R., Jentzsch, T., Wilson, J. R. F., Moghaddamjou, A., Jiang, F., Rienmueller, A., Fehlings, M. G. (2021). Inter-rater Reliability of the Modified Japanese Orthopedic Association Score in Degenerative Cervical Myelopathy: A Cross-sectional Study. Spine (Phila Pa 1976), 46(16), 1063-1069. doi:10.1097/BRS.0000000000003956
88. Merali, Z. G., Witiw, C. D., Badhiwala, J. H., Wilson, J. R., & Fehlings, M. G. (2019). Using a machine learning approach to predict outcome after surgery for degenerative cervical myelopathy. PLoS One, 14(4), e0215133. doi:10.1371/journal.pone.0215133
89. Nagata, K., Ohashi, T., Abe, J., Morita, M., & Inoue, A. (1996). Cervical myelopathy in elderly patients: clinical results and MRI findings before and after decompression surgery. Spinal Cord, 34(4), 220-226. doi:10.1038/sc.1996.41
90. Nagoshi, N., Tetreault, L., Nakashima, H., Arnold, P. M., Barbagallo, G., Kopjar, B., & Fehlings, M. G. (2017). Risk Factors for and Clinical Outcomes of Dysphagia After Anterior Cervical Surgery for Degenerative Cervical Myelopathy: Results from the AOSpine International and North America Studies. J Bone Joint Surg Am, 99(13), 1069-1077. doi:10.2106/JBJS.16.00325
91. Nagoshi, N., Tetreault, L. A., Nakashima, H., Nouri, A., Arnold, P., Zileli, M., Fehlings, M. G. (2016). Do Caucasians and East Asians have Different Outcomes Following Surgery for the Treatment of Degenerative Cervical Myelopathy?: Results From the Prospective Multicenter AOSpine International Study. Spine (Phila Pa 1976), 41(18), 1428-1435. doi:10.1097/BRS.0000000000001555
92. Nakanishi, K., Tanaka, N., Kamei, N., Hiramatsu, T., Ujigo, S., Sumiyoshi, N., Ochi, M. (2015). Electrophysiological assessments of the motor pathway in diabetic patients with compressive cervical myelopathy. J Neurosurg Spine, 23(6), 707-714. doi:10.3171/2015.3.SPINE141060
93. Nakashima, H., Tetreault, L., Nagoshi, N., Nouri, A., Arnold, P., Yukawa, Y., Fehlings, M. G. (2016a). Comparison of Outcomes of Surgical Treatment for Ossification of the Posterior Longitudinal Ligament Versus Other Forms of Degenerative Cervical Myelopathy: Results from the Prospective, Multicenter AOSpine CSM-International Study of 479 Patients. J Bone Joint Surg Am, 98(5), 370-378. doi:10.2106/JBJS.O.00397
94. Nakashima, H., Tetreault, L. A., Nagoshi, N., Nouri, A., Kopjar, B., Arnold, P. M., Fehlings, M. G. (2016). Does age affect surgical outcomes in patients with degenerative cervical myelopathy? Results from the prospective multicenter AOSpine International study on 479 patients. J Neurol Neurosurg Psychiatry, 87(7), 734-740. doi:10.1136/jnnp-2015-311074
95. Ninomiya, K., Shiraishi, T., Aoyama, R., Nori, S., Yamane, J., Kitamura, K., Anazawa, U. (2020). Analysis of the impact of spinopelvic radiographic parameters on the severity of cervical spondylotic myelopathy. J Orthop Sci, 25(6), 966-974. doi:10.1016/j.jos.2020.01.006
96. Ogawa, Y., Chiba, K., Matsumoto, M., Nakamura, M., Takaishi, H., & Toyama, Y. (2006). Postoperative factors affecting neurological recovery after surgery for cervical spondylotic myelopathy. J Neurosurg Spine, 5(6), 483-487. doi:10.3171/spi.2006.5.6.483
97. Ost, K., Jacobs, W. B., Evaniew, N., Cohen-Adad, J., Anderson, D., & Cadotte, D. W. (2021). Spinal Cord Morphology in Degenerative Cervical Myelopathy Patients; Assessing Key Morphological Characteristics Using Machine Vision Tools. J Clin Med, 10(4). doi:10.3390/jcm10040892
98. Ozawa, H., Aizawa, T., Tateda, S., Hashimoto, K., Kanno, H., & Ishizuka, M. (2018). Spinal Cord Swelling After Surgery in Cervical Spondylotic Myelopathy: Relationship With Intramedullary Gd-DTPA Enhancement on MRI. Clin Spine Surg, 31(7), E363-E367. doi:10.1097/BSD.0000000000000664
99. Pasha, I. F., Khalique, A. B., Qureshi, M. A., Talha, M., Farooq, M., Afzal, W., Ahmad, N. (2015). Outcome of surgical treatment of cervical spondylotic myelopathy: Experience in 120 patients. J Pak Med Assoc, 65(11 Suppl 3), S72-76.

100. Peng, J., Zhan, Y. L., Liu, Y. J., Zong, Y., & Xu, J. G. (2019). Plasma VDBP, 25(OH)D, and GSH levels predict surgical outcome in patients with cervical spondylotic myelopathy. Kaohsiung J Med Sci, 35(2), 102-110. doi:10.1002/kjm2.12015
101. Phillips, F. M., Geisler, F. H., Gilder, K. M., Reah, C., Howell, K. M., & McAfee, P. C. (2015). Long-term Outcomes of the US FDA IDE Prospective, Randomized Controlled Clinical Trial Comparing PCM Cervical Disc Arthroplasty With Anterior Cervical Discectomy and Fusion. Spine (Phila Pa 1976), 40(10), 674-683. doi:10.1097/BRS.0000000000000869
102. Phillips, F. M., Lee, J. Y., Geisler, F. H., Cappuccino, A., Chaput, C. D., DeVine, J. G., McAfee, P. C. (2013). A prospective, randomized, controlled clinical investigation comparing PCM cervical disc arthroplasty with anterior cervical discectomy and fusion. 2-year results from the US FDA IDE clinical trial. Spine (Phila Pa 1976), 38(15), E907-918. doi:10.1097/BRS.0b013e318296232f
103. Pope, D. H., Mowforth, O. D., Davies, B. M., & Kotter, M. R. N. (2020). Diagnostic Delays Lead to Greater Disability in Degenerative Cervical Myelopathy and Represent a Health Inequality. Spine (Phila Pa 1976), 45(6), 368-377. doi:10.1097/BRS.0000000000003305
104. Rajshekhar, V., & Muliyil, J. (2007). Patient perceived outcome after central corpectomy for cervical spondylotic myelopathy. Surg Neurol, 68(2), 185-190; discussion 190-181. doi:10.1016/j.surneu.2006.10.071
105. Riew, K. D., Buchowski, J. M., Sasso, R., Zdeblick, T., Metcalf, N. H., & Anderson, P. A. (2008). Cervical disc arthroplasty compared with arthrodesis for the treatment of myelopathy. J Bone Joint Surg Am, 90(11), 2354-2364. doi:10.2106/JBJS.G.01608
106. Romagna, A., Wilson, J. R., Jacobs, W. B., Johnson, M. G., Bailey, C. S., Christie, S., Dea, N. (2020). Factors Associated With Return to Work After Surgery for Degenerative Cervical Spondylotic Myelopathy: Cohort Analysis From the Canadian Spine Outcomes and Research Network. Global Spine J, 2192568220958669. doi:10.1177/2192568220958669
107. Sasso, R. C., Anderson, P. A., Riew, K. D., & Heller, J. G. (2011). Results of cervical arthroplasty compared with anterior discectomy and fusion: four-year clinical outcomes in a prospective, randomized controlled trial. J Bone Joint Surg Am, 93(18), 1684-1692. doi:10.2106/JBJS.J.00476
108. Sebastián, C., Raya, J. P., Ortega, M., Olalla, E., Lemos, V., & Romero, R. (1997). Intraoperative control by somatosensory evoked potentials in the treatment of cervical myeloradiculopathy. Results in 210 cases. Eur Spine J, 6(5), 316-323. doi:10.1007/BF01142677
109. Seng, C., Tow, B. P., Siddiqui, M. A., Srivastava, A., Wang, L., Yew, A. K., Yue, W. M. (2013). Surgically treated cervical myelopathy: a functional outcome comparison study between multilevel anterior cervical decompression fusion with instrumentation and posterior laminoplasty. Spine J, 13(7), 723-731. doi:10.1016/j.spinee.2013.02.038
110. Shen, C., Xu, H., Xu, B., Zhang, X., Li, X., Yang, Q., & Ma, X. (2018). Value of conventional MRI and diffusion tensor imaging parameters in predicting surgical outcome in patients with degenerative cervical myelopathy. J Back Musculoskelet Rehabil, 31(3), 525-532. doi:10.3233/BMR-170972
111. Shigematsu, H., Ueda, Y., Koizumi, M., Takeshima, T., Tanaka, Y., Satoh, N., Takakura, Y. (2008). Does developmental canal stenosis influence surgical results of bilateral open-door laminoplasty for cervical spondylotic myelopathy? J Neurosurg Spine, 9(4), 358-362. doi:10.3171/SPI.2008.9.10.358
112. Shin, J. J., Jin, B. H., Kim, K. S., Cho, Y. E., & Cho, W. H. (2010). Intramedullary high signal intensity and neurological status as prognostic factors in cervical spondylotic myelopathy. Acta Neurochir (Wien), 152(10), 1687-1694. doi:10.1007/s00701-010-0692-8
113. Singh, A., & Crockard, H. A. (2001). Comparison of seven different scales used to quantify severity of cervical spondylotic myelopathy and post-operative improvement. J Outcome Meas, 5(1), 798-818.
114. Singh, A., Gnanalingham, K., Casey, A., & Crockard, A. (2006). Quality of life assessment using the Short Form-12 (SF-12) questionnaire in patients with cervical spondylotic myelopathy: comparison with SF-36. Spine (Phila Pa 1976), 31(6), 639-643. doi:10.1097/01.brs.0000202744.48633.44
115. Singrakhia, M. D., Malewar, N. R., Singrakhia, S. M., & Deshmukh, S. S. (2017). Cervical Laminectomy with Lateral Mass Screw Fixation in Cervical Spondylotic Myelopathy: Neurological and Sagittal Alignment Outcome: Do We Need Lateral Mass Screws at each Segment? Indian J Orthop, 51(6), 658-665. doi:10.4103/ortho.IJOrtho_266_16
116. Su, N., Fei, Q., Wang, B. Q., Kang, N., Zhang, Q. M., Tang, H. H., Yang, Y. (2019). Comparison of clinical outcomes of expansive open-door laminoplasty with unilateral or bilateral fixation and fusion for treating cervical spondylotic myelopathy: a multi-center prospective study. BMC Surg, 19(1), 116. doi:10.1186/s12893-019-0583-8
117. Suri, A., Chabbra, R. P., Mehta, V. S., Gaikwad, S., & Pandey, R. M. (2003). Effect of intramedullary signal changes on the surgical outcome of patients with cervical spondylotic myelopathy. Spine J, 3(1), 33-45. doi:10.1016/s1529-9430(02)00448-5

118. Tetreault, L., Kopjar, B., Côté, P., Arnold, P., & Fehlings, M. G. (2015). A Clinical Prediction Rule for Functional Outcomes in Patients Undergoing Surgery for Degenerative Cervical Myelopathy: Analysis of an International Prospective Multicenter Data Set of 757 Subjects. J Bone Joint Surg Am, 97(24), 2038-2046. doi:10.2106/JBJS.O.00189
119. Tetreault, L., Kopjar, B., Nouri, A., Arnold, P., Barbagallo, G., Bartels, R., Fehlings, M. G. (2017). The modified Japanese Orthopaedic Association scale: establishing criteria for mild, moderate and severe impairment in patients with degenerative cervical myelopathy. Eur Spine J, 26(1), 78-84. doi:10.1007/s00586-016-4660-8
120. Tetreault, L., Nagoshi, N., Nakashima, H., Singh, A., Kopjar, B., Arnold, P., & Fehlings, M. G. (2017). Impact of Depression and Bipolar Disorders on Functional and Quality of Life Outcomes in Patients Undergoing Surgery for Degenerative Cervical Myelopathy: Analysis of a Combined Prospective Dataset. Spine (Phila Pa 1976), 42(6), 372-378. doi:10.1097/BRS.0000000000001777
121. Tetreault, L., Nouri, A., Kopjar, B., Côté, P., & Fehlings, M. G. (2015). The Minimum Clinically Important Difference of the Modified Japanese Orthopaedic Association Scale in Patients with Degenerative Cervical Myelopathy. Spine (Phila Pa 1976), 40(21), 1653-1659. doi:10.1097/BRS.0000000000001127
122. Tetreault, L., Tan, G., Kopjar, B., Côté, P., Arnold, P., Nugaeva, N., Fehlings, M. G. (2016). Clinical and Surgical Predictors of Complications Following Surgery for the Treatment of Cervical Spondylotic Myelopathy: Results From the Multicenter, Prospective AOSpine International Study of 479 Patients. Neurosurgery, 79(1), 33-44. doi:10.1227/NEU.0000000000001151
123. Tetreault, L., Wilson, J. R., Kotter, M. R., Nouri, A., Côté, P., Kopjar, B., Fehlings, M. G. (2016). Predicting the minimum clinically important difference in patients undergoing surgery for the treatment of degenerative cervical myelopathy. Neurosurg Focus, 40(6), E14. doi:10.3171/2016.3.FOCUS1665
124. Tetreault, L., Wilson, J. R., Kotter, M. R. N., Côté, P., Nouri, A., Kopjar, B., Fehlings, M. G. (2019). Is Preoperative Duration of Symptoms a Significant Predictor of Functional Outcomes in Patients Undergoing Surgery for the Treatment of Degenerative Cervical Myelopathy? Neurosurgery, 85(5), 642-647. doi:10.1093/neuros/nyy474
125. Tetreault, L. A., Côté, P., Kopjar, B., Arnold, P., Fehlings, M. G., & Network, A. N. A. a. I. C. T. R. (2015a). A clinical prediction model to assess surgical outcome in patients with cervical spondylotic myelopathy: internal and external validations using the prospective multicenter AOSpine North American and international datasets of 743 patients. Spine J, 15(3), 388-397. doi:10.1016/j.spinee.2014.12.145
126. Tetreault, L. A., Kopjar, B., Vaccaro, A., Yoon, S. T., Arnold, P. M., Massicotte, E. M., & Fehlings, M. G. (2013). A clinical prediction model to determine outcomes in patients with cervical spondylotic myelopathy undergoing surgical treatment: data from the prospective, multi-center AOSpine North America study. J Bone Joint Surg Am, 95(18), 1659-1666. doi:10.2106/JBJS.L.01323
127. Tetreault, L. A., Zhu, M. P., Howard, R. M., Sorefan-Mangou, F., Patel, A. A., Schroeder, G. D., Wilson, J. R. (2019). The discrepancy between functional outcome and self-reported health status after surgery for degenerative cervical myelopathy. Spine J, 19(11), 1809-1815. doi:10.1016/j.spinee.2019.06.026
128. Vedantam, A., & Rajshekhar, V. (2016). Clinical adjacent-segment pathology after central corpectomy for cervical spondylotic myelopathy: incidence and risk factors. Neurosurg Focus, 40(6), E12. doi:10.3171/2016.2.FOCUS1626
129. Wan, J., Xu, T. T., Shen, Q. F., Li, H. N., & Xia, Y. P. (2011). Influence of hinge position on the effectiveness of open-door expansive laminoplasty for cervical spondylotic myelopathy. Chin J Traumatol, 14(1), 36-41.
130. Wang, L., Wei, F., Liu, S., Wan, Y., Chen, N., Cui, S., Huang, Y. (2015). Can Modified Kurokawa's Double-Door Laminoplasty Reduce the Incidence of Axial Symptoms at Long-term Follow-up?: A Prospective Study of 152 Patients With Cervical Spondylotic Myelopathy. J Spinal Disord Tech, 28(4), E186-193. doi:10.1097/BSD.0000000000000242
131. Wang, Z., Sakakibara, T., & Kasai, Y. (2014). Overactive bladder in cervical spondylotic myelopathy. J Orthop Sci, 19(1), 22-25. doi:10.1007/s00776-013-0491-1
132. Whitmore, R. G., Ghogawala, Z., Petrov, D., Schwartz, J. S., & Stein, S. C. (2013). Functional outcome instruments used for cervical spondylotic myelopathy: interscale correlation and prediction of preference-based quality of life. Spine J, 13(8), 902-907. doi:10.1016/j.spinee.2012.11.058
133. Wilson, J. R., Tetreault, L. A., Schroeder, G., Harrop, J. S., Prasad, S., Vaccaro, A., Fehlings, M. G. (2017). Impact of Elevated Body Mass Index and Obesity on Long-term Surgical Outcomes for Patients With Degenerative Cervical Myelopathy: Analysis of a Combined Prospective Dataset. Spine (Phila Pa 1976), 42(3), 195-201. doi:10.1097/BRS.0000000000001859
134. Wilson, J. R. F., Badhiwala, J. H., Jiang, F., Wilson, J. R., Kopjar, B., Vaccaro, A. R., & Fehlings, M. G. (2019). The Impact of Older Age on Functional Recovery and Quality of Life Outcomes after Surgical Decompression for Degenerative Cervical Myelopathy: Results from an Ambispective, Propensity-Matched Analysis from the CSM-NA and CSM-I International, Multi-Center Studies. J Clin Med, 8(10). doi:10.3390/jcm8101708

135. Witiw, C. D., Tetreault, L. A., Smieliauskas, F., Kopjar, B., Massicotte, E. M., & Fehlings, M. G. (2017). Surgery for degenerative cervical myelopathy: a patient-centered quality of life and health economic evaluation. Spine J, 17(1), 15-25. doi:10.1016/j.spinee.2016.10.015
136. Xu, J., Zhou, X., Xu, C., Ding, X., Jin, K., Yan, M., Yuan, W. (2020). Clinical study on improving postoperative symptoms of cervical spondylotic myelopathy by Qishe pill. Medicine (Baltimore), 99(36), e21994. doi:10.1097/MD.0000000000021994
137. Xu, N., Zhang, Y., Zhou, G., Zhao, Q., & Wang, S. (2020). The value of dynamic MRI in the treatment of cervical spondylotic myelopathy: a protocol for a prospective randomized clinical trial. BMC Musculoskelet Disord, 21(1), 83. doi:10.1186/s12891-020-3106-y
138. Yamada, K., Suda, K., Matsumoto Harmon, S., Komatsu, M., Ushiku, C., Takahata, M., Iwasaki, N. (2019). Rapidly progressive cervical myelopathy had a high risk of developing deep venous thrombosis: a prospective observational study in 289 cases with degenerative cervical spine disease. Spinal Cord, 57(1), 58-64. doi:10.1038/s41393-018-0213-9
139. Yang, L., Yang, C., Pang, X., Li, D., Chen, X., Shi, J., Peng, B. (2017). Cervical Decompression Surgery for Cervical Spondylotic Myelopathy and Concomitant Hypertension: A Multicenter Prospective Cohort Study. Spine (Phila Pa 1976), 42(12), 903-908. doi:10.1097/BRS.0000000000001941
140. Ying, Z., Xinwei, W., Jing, Z., Shengming, X., Bitao, L., Tao, Z., & Wen, Y. (2007). Cervical corpectomy with preserved posterior vertebral wall for cervical spondylotic myelopathy: a randomized control clinical study. Spine (Phila Pa 1976), 32(14), 1482-1487. doi:10.1097/BRS.0b013e318068b30a
141. Yoshida, G., Kanemura, T., Ishikawa, Y., Matsumoto, A., Ito, Z., Tauchi, R., Ishiguro, N. (2013). The effects of surgery on locomotion in elderly patients with cervical spondylotic myelopathy. Eur Spine J, 22(11), 2545-2551. doi:10.1007/s00586-013-2961-8
142. Yukawa, Y., Kato, F., Ito, K., Horie, Y., Hida, T., Machino, M., Matsuyama, Y. (2008). Postoperative changes in spinal cord signal intensity in patients with cervical compression myelopathy: comparison between preoperative and postoperative magnetic resonance images. J Neurosurg Spine, 8(6), 524-528. doi:10.3171/SPI/2008/8/6/524
143. Yukawa, Y., Kato, F., Yoshihara, H., Yanase, M., & Ito, K. (2007). MR T2 image classification in cervical compression myelopathy: predictor of surgical outcomes. Spine (Phila Pa 1976), 32(15), 1675-1678; discussion 1679. doi:10.1097/BRS.0b013e318074d62e
144. Zhang, J. T., Meng, F. T., Wang, S., Wang, L. F., & Shen, Y. (2015). Predictors of surgical outcome in cervical spondylotic myelopathy: focusing on the quantitative signal intensity. Eur Spine J, 24(12), 2941-2945. doi:10.1007/s00586-015-4109-5
145. Zhang, J. T., Wang, L. F., Wang, S., Li, J., & Shen, Y. (2016). Risk factors for poor outcome of surgery for cervical spondylotic myelopathy. Spinal Cord, 54(12), 1127-1131. doi:10.1038/sc.2016.64
146. Zhang, X., Chen, C., Zhang, Y., Wang, Z., Wang, B., Yan, W., Wang, Y. (2012). Randomized, controlled, multicenter, clinical trial comparing BRYAN cervical disc arthroplasty with anterior cervical decompression and fusion in China. Spine (Phila Pa 1976), 37(6), 433-438. doi:10.1097/BRS.0b013e31822699fa
147. Zhang, Y., Zhou, F., & Sun, Y. (2015). Assessment of health-related quality of life using the SF-36 in Chinese cervical spondylotic myelopathy patients after surgery and its consistency with neurological function assessment: a cohort study. Health Qual Life Outcomes, 13, 39. doi:10.1186/s12955-015-0237-1
148. Zhou, F., Zhang, Y., Sun, Y., Zhang, F., Pan, S., & Liu, Z. (2015). Assessment of the minimum clinically important difference in neurological function and quality of life after surgery in cervical spondylotic myelopathy patients: a prospective cohort study. Eur Spine J, 24(12), 2918-2923. doi:10.1007/s00586-015-4208-3
149. Zong, Y., Xue, Y., Zhao, Y., Ding, H., He, D., Li, Z., Wang, Y. (2014). Depression contributed an unsatisfactory surgery outcome among the posterior decompression of the cervical spondylotic myelopathy patients: a prospective clinical study. Neurol Sci, 35(9), 1373-1379. doi:10.1007/s10072-014-1714-8
